# Update on the Laboratory Diagnosis of Invasive Fungal Infections

**DOI:** 10.4084/MJHID.2011.002

**Published:** 2011-01-14

**Authors:** Brunella Posteraro, Riccardo Torelli, Elena De Carolis, Patrizia Posteraro, Maurizio Sanguinetti

**Affiliations:** 1 Institute of Microbiology, Università Cattolica del Sacro Cuore, Largo F. Vito, 1-00168, Rome, Italy; 2 Laboratory of Clinical Pathology and Microbiology, Ospedale San Carlo-IDI IRCSS, via Aurelia, 275-00165 Rome, Italy

## Abstract

Recent advances in the management of patients with haematological malignancies and transplant recipients have paralleled an increase in the incidence of fungal diseases due to pathogenic genera such as *Candida* and *Aspergillus* and the emergence of less common genera including *Fusarium* and *Zygomycetes*. Despite availability of new antifungal agents these opportunistic infections have high mortality. Rapid and reliable species identification is essential for antifungal treatment, but detection of the increasing diversity of fungal pathogens by conventional phenotypic methods remains difficult and time-consuming, and the results may sometimes be inconclusive, especially for unusual species. New diagnostic techniques (e.g., 1,3-beta-d-glucan detection) could improve this scenario, although further studies are necessary to confirm their usefulness in clinical practice.

## Introduction

Despite the development of new techniques and new antifungal agents, diagnosis of invasive fungal infection (IFI), which still relies upon a combination of clinical observation and laboratory investigation, remains a challenge especially for immunocompromised patients with haematological disease.^1^ This has important clinical repercussions since delayed diagnosis and therapy contribute significantly to the high mortality rates associated with IFIs,^2^ whereas early intervention with antifungal drugs may result in more effective management of high-risk patients.^3^ While superficial and subcutaneous fungal infections often produce characteristic lesions that suggest the diagnosis, a thorough knowledge of potential causative organisms is yet required to aid the diagnostic process, mainly in situations where systemic fungal infection is suspected but the clinical presentation is nonspecific and then ascribable to a wide range of infections, underlying illnesses, or complication of treatments.^4^

The exact identification of the infecting organism is became essential in light of the increased use of prophylactic schedules that predispose the patient not just to fungal infection, but also to the selection of fungal species such as non-*albicans Candida* (e.g., *C. glabrata* and *C. krusei*), *Aspergillus terreus*, *Scedosporium* species, and *Zygomycetes*, many of which are intrinsically resistant to the available antifungal agents.^5^,^6^

## Culture-based detection methods

Laboratory diagnosis of IFI remains based on conventional approaches, such as the direct microscopically detection of the etiologic agent in clinical specimen and the isolation and identification of the pathogen in culture, and non-culture based methods involving detection of a serologic response to the pathogen or other marker of its presence such as fungal antigens or metabolites.^4^ Visual examination of fungi in tissue samples allows presumptive identification based on cellular morphology and staining properties, but it should be appreciated that invasive procedures necessary to obtain biopsies may be precluded in haematological patients. It should be noted that microbiological cultures are often insensitive or of limited use, since even with modern blood cultures systems candidaemia can be transient and not detected, or *Aspergillus* cannot be cultured from a significant proportion of sputum or bronchoalveolar lavage samples from patients with invasive aspergillosis (IA).^7^

Although a variety of culture media and incubation conditions may be required for recovery of fungal agents, chromogenic primary isolation media (e.g., CHROMagar Candida medium) can be employed for the presumptive identification of the most medically important *Candida* species, including *C. albicans*, *C. tropicalis*, *C. glabrata*, and *C. parapsilosis*. Most yeasts isolated from clinical samples can be identified using one of the numerous commercial identification systems, such as API 20C AUX, VITEK 2, and RapID Yeast Plus.^4^,^8^ Although the kits are relatively easy to use, it should be remembered that additional morphologic-based tests are often required to avoid confusion between organisms with identical biochemical profiles.

Unlike pathogenic yeasts, filamentous fungi can be identified only by visualization of macroscopic (colonial form, surface colour, and pigmentation) or microscopic (spore-bearing structures) morphologic characteristics, following to sub-cultivation of a mould isolate to encourage sporulation,^4^ a process that takes days to weeks. In addition to the use of genetic probes for the culture confirmation of dimorphic systemic fungal pathogens (e.g., *Histoplasma capsulatum*), an alternative and useful approach to the detection and identification of fungi in clinical specimens involves a broad-range polymerase chain reaction (PCR) followed by nucleic acid sequencing, after which the nucleic acid sequence is compared with known sequence database and identification is based on DNA homology.^9^ However, these methods are expensive and time-consuming, and they are not currently suitable for routine identification.

By contrast, matrix-assisted laser-desorption/ionization time-of-flight mass spectrometry (MALDI-TOF MS) is becoming a reliable method for identification of microorganisms. The remarkable reproducibility of the methodology is based on the measurement of constantly expressed and highly abundant proteins such as ribosomal molecules.^10^,^11^ Some recent studies have shown the potential of the MALDI-TOF technique to identify fungal clinical species such as *Aspergillus* and *Fusarium*. Recently, the MALDI-TOF technology provided a fast and accurate identification of common and unusual species of *Aspergillus* when tested on 124 clinical and 16 environmental isolates.^12^ With regard to *Fusarium* species, a recent case report supported the usefulness of MALDI-TOF analysis in diagnosing an infection due to *Fusarium proliferatum*, which is a very infrequent pathogen within this genus.^13^

## Antigen-based detection methods

To facilitate early diagnosis of IFI, important advances have been made in the development of laboratory markers (e.g., galactomannan [GM] and 1,3-beta-d-glucan [BG] assays), which have led to the potential for newer paradigms regarding prevention and early treatment of IFIs.^14^ Among fungal markers, GM, a component of fungal cell wall that can be detected by a sandwich-type enzyme-linked immunosorbent assay (ELISA) in serum or plasma,^15^ bronchoalveolar lavage (BAL) fluid,^16^,^17^ and cerebrospinal fluid,^18^ has recently been approved by the US Food and Drug Administration at a serum cut-off of 0.5, as a diagnostic adjunct for IA.^14^ Together with clinical criteria, a positive serum or BAL fluid GM would strongly suggest probable IA, as defined by authoritative consensus criteria.^19^ However, this technique has shown contradictory results, in terms of sensitivity and specificity, due to several factors, including the impact of prior antifungal therapy on the levels of circulating fungal components,^20^ the occurrence of false-positive results in association with some antibiotic treatments,^21^,^22^ and the different cutoffs of positivity among studies.^14^ Thus, a recently published meta-analysis of 27 studies showed an overall sensitivity of 71% and specificity of 89% for proven cases of IA when used for surveillance.^23^ In addition, GM also correlates well with outcome.^24^

In contrast to GM, BG is a cell wall constituent of several fungi, including *Aspergillus*, *Candida*, and *Fusarium*, a spectrum of pathogens that encompasses the majority of those emerging in neutropenic patients with either prolonged neutropenia or chemotherapy-induced mucositis, with some notable exceptions such as *Zygomycetes*, a rare but emerging cause of invasive mycosis.^25^,^26^ Measurement of serum BG has been shown to be an aid in the diagnosis of fungaemia and deep-seated mycoses, including IA.^27^,^28^ Among commercially available assays, the Fungitell, which is also an ELISA technique, is widely used to detect serum BG concentrations as low as 1 pg/mL.^27^ The cutoff for a positive result is >80 pg/ml. As with GM, variable results have been reported for BG assay, with a slightly higher sensitivity and specificity, ranging from 70% to 90%.^28^,^29^ When performances of both GM and BD tests were compared to determine their diagnostic usefulness for high-risk haematological malignancy patients, GM assay was significantly better for detecting non-*fumigatus Aspergillus* species, whereas BG was shown to have a higher sensitivity in detecting IA and other mould infections.^30^

Non-culture based methods for diagnosis of candidiasis are of limited value because the levels of circulating antigens are low and the transient nature of the antigenaemia requires sensitive assays and frequent sampling of at-risk patients.^1^,^31^ However, the use of Platelia Candida, an ELISA that combines the detection of mannan antigen and anti-mannan antibodies in serum, led to earlier diagnosis of *Candida* infection when compared with blood cultures.^31^ In haematological patients with hepatosplenic lesions, assessing mannan/anti-mannan antibodies shortened significantly the median time of diagnosis of candidiasis when compared with imaging.^32^

## Molecular-based detection methods

A range of polymerase chain reaction (PCR)-based methods have been developed with the prospect of give highly specific, highly sensitive, and rapid means for fungal detection and identification.^33^ Most of them have focused on *Aspergillus* and *Candida* species, using different specimens types (e.g., serum, plasma, or BAL fluid), even though pan-fungal PCR amplification technology may be able to detect a broad range of fungal targets.^33^ Although PCR has been studied for years, the lack of standardization and clinical validation has led to its exclusion from consensus criteria for defining IFI.^19^ Nevertheless, a recent prospective evaluation of serial PCR assays against^34^ or along with GM and computed tomography^35^ was carried out in haematological patients, thus showing acceptable sensitivity and specificity. In such one study, the combination of serial PCR and GM detected 100% of aspergillosis cases, with a positive predictive value of 75.1%.^35^ Of note, in a systematic review and meta-analysis of *Aspergillus* PCR tests for diagnosis of IA,^36^ the authors proposed that a single PCR-negative test is sufficient to exclude IA, whereas two PCR-positive results are required to confirm disease. Compared with *Aspergillus* PCR, only a few *Candida* PCR methods have received major clinical evaluation.^37^ As confirmed by a national consensus evaluation,^38^ performance of these tests is generally good, with sensitivities and specificities consistently >90%.^33^ Although addressed to critically ill patients, a prospective clinical trial published in 2008 reported positive predictive values and negative predictive values of >90% for a PCR method that detects several species of *Candida*.^39^

## Conclusion

Molecular detection methods, combined with additional microbiological and clinical information, has the potential not only to accurately and rapidly identify fungal pathogens, but also to indicate whether the pathogen is likely to respond to conventional antifungal treatment.^9^ Inclusion of these methods in a diagnostic surveillance strategy to exclude IFI in high-risk patients with haematological malignancy^40^ should result in improved clinical management, thus allowing more rational use of antifungal drugs.

## References

[1] Cuenca-Estrella M, Bernal-Martinez L, Buitrago MJ, Castelli MV, Gomez-Lopez A, Zaragoza O, Rodriguez-Tudela JL (2008). Update on the epidemiology and diagnosis of invasive fungal infection. Int J Antimicrob Agents.

[2] Morrell M, Fraser VJ, Kollef MH (2005). Delaying the empiric treatment of Candida bloodstream infection until positive blood culture results are obtained: a potential risk factor for hospital mortality. Antimicrob Agents Chemother.

[3] Murali S, Langston A (2009). Advances in antifungal prophylaxis and empiric therapy in patients with hematologic malignancies. Transpl Infect Dis.

[4] Dismukes WE, Pappas PG, Sobel JD (2003). Clinical Mycology.

[5] Perea S, Patterson TF (2002). Antifungal resistance in pathogenic fungi. Clin Infect Dis.

[6] Rodriguez-Tudela JL, Alcazar-Fuoli L, Cuesta I, Alastruey-Izquierdo A, Monzon A, Mellado E, Cuenca-Estrella M (2008). Clinical relevance of resistance to antifungals. Int J Antimicrob Agents.

[7] Singh N, Paterson DL (2005). Aspergillus infections in transplant recipients. Clin Microbiol Rev.

[8] Sanguinetti M, Porta R, Sali M, La Sorda M, Pecorini G, Fadda G, Posteraro B (2007). Evaluation of VITEK 2 and RapID yeast plus systems for yeast species identification: experience at a large clinical microbiology laboratory. J Clin Microbiol.

[9] Persing DH, Tenover FC, Versalovic J, Tang JW, Unger ER, Relman DA, White TJ (2004). Molecular Microbiology.

[10] Fenselau C, Demirev PA (2001). Characterization of intact microorganisms by MALDI mass spectrometry. Mass Spectrom Rev.

[11] Santos C, Paterson RR, Venâncio A, Lima N (2010). Filamentous fungal characterizations by matrix-assisted laser desorption/ionization time-of-flight mass spectrometry. J Appl Microbiol.

[12] Alanio A, Beretti JL, Dauphin B, Mellado E, Quesne G, Lacroix C, Amara A, Berche P, Nassif X, Bougnoux ME (2010). MALDI-TOF Mass Spectrometry for fast and accurate identification of clinically relevant Aspergillus species. Clin Microbiol Infect.

[13] Seyfarth F, Ziemer M, Sayer HG, Burmester A, Erhard M, Welker M, Schliemann S, Straube E, Hipler UC (2008). The use of ITS DNA sequence analysis and MALDI-TOF mass spectrometry in diagnosing an infection with Fusarium proliferatum. Exp Dermatol.

[14] Almyroudis NG, Segal BH (2009). Prevention and treatment of invasive fungal diseases in neutropenic patients. Curr Opin Infect Dis.

[15] Maertens J, Verhaegen J, Lagrou K, Van Eldere J, Boogaerts M (2001). Screening for circulating galactomannan as a noninvasive diagnostic tool for invasive aspergillosis in prolonged neutropenic patients and stem cell transplantation recipients: a prospective validation. Blood.

[16] Sanguinetti M, Posteraro B, Pagano L, Pagliari G, Fianchi L, Mele L, La Sorda M, Franco A, Fadda G (2003). Comparison of real-time PCR, conventional PCR, and galactomannan antigen detection by enzyme-linked immunosorbent assay using bronchoalveolar lavage fluid samples from hematology patients for diagnosis of invasive pulmonary aspergillosis. J Clin Microbiol.

[17] Clancy CJ, Jaber RA, Leather HL, Wingard JR, Staley B, Wheat LJ, Cline CL, Rand KH, Schain D, Baz M, Nguyen MH (2007). Bronchoalveolar lavage galactomannan in diagnosis of invasive pulmonary aspergillosis among solid-organ transplant recipients. J Clin Microbiol.

[18] Viscoli C, Machetti M, Gazzola P, De Maria A, Paola D, Van Lint MT, Gualandi F, Truini M, Bacigalupo A (2002). Aspergillus galactomannan antigen in the cerebrospinal fluid of bone marrow transplant recipients with probable cerebral aspergillosis. J Clin Microbiol.

[19] De Pauw B, Walsh TJ, Donnelly JP, Stevens DA, Edwards JE, Calandra T, Pappas PG, Maertens J, Lortholary O, Kauffman CA, Denning DW, Patterson TF, Maschmeyer G, Bille J, Dismukes WE, Herbrecht R, Hope WW, Kibbler CC, Kullberg BJ, Marr KA, Munoz P, Odds FC, Perfect JR, Restrepo A, Ruhnke M, Segal BH, Sobel JD, Sorrell TC, Viscoli C, Wingard JR, Zaoutis T, Bennett JE, European Organization for Research and Treatment of Cancer/Invasive Fungal Infections Cooperative Group; National Institute of Allergy and Infectious Diseases Mycoses Study Group (EORTC/MSG) Consensus Group (2008). Revised definitions of invasive fungal disease from the European Organization for Research and Treatment of Cancer/Invasive Fungal Infections Cooperative Group and the National Institute of Allergy and Infectious Diseases Mycoses Study Group (EORTC/MSG) Consensus Group. Clin Infect Dis.

[20] Marr KA, Laverdiere M, Gugel A, Leisenring W (2005). Antifungal therapy decreases sensitivity of the Aspergillus galactomannan enzyme immunoassay. Clin Infect Dis.

[21] Adam O, Aupérin A, Wilquin F, Bourhis JH, Gachot B, Chachaty E (2004). Treatment with piperacillin-tazobactam and false-positive Aspergillus galactomannan antigen test results for patients with hematological malignancies. Clin Infect Dis.

[22] Mattei D, Rapezzi D, Mordini N, Cuda F, Lo Nigro C, Musso M, Arnelli A, Cagnassi S, Gallamini A (2004). False-positive Aspergillus galactomannan enzyme-linked immunosorbent assay results in vivo during amoxicillin-clavulanic acid treatment. J Clin Microbiol.

[23] Pfeiffer CD, Fine JP, Safdar N (2006). Diagnosis of invasive aspergillosis using a galactomannan assay: a meta-analysis. Clin Infect Dis.

[24] Miceli MH, Grazziutti ML, Woods G, Zhao W, Kocoglu MH, Barlogie B, Anaissie E (2008). Strong correlation between serum aspergillus galactomannan index and outcome of aspergillosis in patients with hematological cancer: clinical and research implications. Clin Infect Dis.

[25] Chamilos G, Luna M, Lewis RE, Bodey GP, Chemaly R, Tarrand JJ, Safdar A, Raad II, Kontoyiannis DP (2006). Invasive fungal infections in patients with hematologic malignancies in a tertiary care cancer center: an autopsy study over a 15-year period (1989–2003). Haematologica.

[26] Pagano L, Caira M, Nosari A, Van Lint MT, Candoni A, Offidani M, Aloisi T, Irrera G, Bonini A, Picardi M, Caramatti C, Invernizzi R, Mattei D, Melillo L, de Waure C, Reddiconto G, Fianchi L, Valentini CG, Girmenia C, Leone G, Aversa F (2007). Fungal infections in recipients of hematopoietic stem cell transplants: results of the SEIFEM B-2004 study--Sorveglianza Epidemiologica Infezioni Fungine Nelle Emopatie Maligne. Clin Infect Dis.

[27] Odabasi Z, Mattiuzzi G, Estey E, Kantarjian H, Saeki F, Ridge RJ, Ketchum PA, Finkelman MA, Rex JH, Ostrosky-Zeichner L (2004). Beta-D-glucan as a diagnostic adjunct for invasive fungal infections: validation, cutoff development, and performance in patients with acute myelogenous leukemia and myelodysplastic syndrome. Clin Infect Dis.

[28] Ostrosky-Zeichner L, Alexander BD, Kett DH, Vazquez J, Pappas PG, Saeki F, Ketchum PA, Wingard J, Schiff R, Tamura H, Finkelman MA, Rex JH (2005). Multicenter clinical evaluation of the (1→3) beta-D-glucan assay as an aid to diagnosis of fungal infections in humans. Clin Infect Dis.

[29] Kami M, Tanaka Y, Kanda Y, Ogawa S, Masumoto T, Ohtomo K, Matsumura T, Saito T, Machida U, Kashima T, Hirai H (2000). Computed tomographic scan of the chest, latex agglutination test and plasma (1→3)-beta-D-glucan assay in early diagnosis of invasive pulmonary aspergillosis: a prospective study of 215 patients. Haematologica.

[30] Hachem RY, Kontoyiannis DP, Chemaly RF, Jiang Y, Reitzel R, Raad I (2009). Utility of galactomannan enzyme immunoassay and (1→3) beta-D-glucan in diagnosis of invasive fungal infections: low sensitivity for Aspergillus fumigatus infection in hematologic malignancy patients. J Clin Microbiol.

[31] Sendid B, Poirot JL, Tabouret M, Bonnin A, Caillot D, Camus D, Poulain D (2002). Combined detection of mannanaemia and antimannan antibodies as a strategy for the diagnosis of systemic infection caused by pathogenic Candida species. J Med Microbiol.

[32] Prella M, Bille J, Pugnale M, Duvoisin B, Cavassini M, Calandra T, Marchetti O (2005). Early diagnosis of invasive candidiasis with mannan antigenemia and antimannan antibodies. Diagn Microbiol Infect Dis.

[33] White PL, Perry MD, Barnes RA (2009). An update on the molecular diagnosis of invasive fungal disease. FEMS Microbiol Lett.

[34] Suarez F, Lortholary O, Buland S, Rubio MT, Ghez D, Mahé V, Quesne G, Poirée S, Buzyn A, Varet B, Berche P, Bougnoux ME (2008). Detection of circulating Aspergillus fumigatus DNA by real-time PCR assay of large serum volumes improves early diagnosis of invasive aspergillosis in high-risk adult patients under hematologic surveillance. J Clin Microbiol.

[35] Cuenca-Estrella M, Meije Y, Diaz-Pedroche C, Gomez-Lopez A, Buitrago MJ, Bernal-Martinez L, Grande C, Juan RS, Lizasoain M, Rodriguez-Tudela JL, Aguado JM (2009). Value of serial quantification of fungal DNA by a real-time PCR-based technique for early diagnosis of invasive aspergillosis in patients with febrile neutropenia. J Clin Microbiol.

[36] Mengoli C, Cruciani M, Barnes RA, Loeffler J, Donnelly JP (2009). Use of PCR for diagnosis of invasive aspergillosis: systematic review and meta-analysis. Lancet Infect Dis.

[37] White PL, Archer AE, Barnes RA (2005). Comparison of non-culture-based methods for detection of systemic fungal infections, with an emphasis on invasive Candida infections. J Clin Microbiol.

[38] White PL, Barton R, Guiver M, Linton CJ, Wilson S, Smith M, Gomez BL, Carr MJ, Kimmitt PT, Seaton S, Rajakumar K, Holyoake T, Kibbler CC, Johnson E, Hobson RP, Jones B, Barnes RA (2006). A consensus on fungal polymerase chain reaction diagnosis?: a United Kingdom-Ireland evaluation of polymerase chain reaction methods for detection of systemic fungal infections. J Mol Diagn.

[39] McMullan R, Metwally L, Coyle PV, Hedderwick S, McCloskey B, O’Neill HJ, Patterson CC, Thompson G, Webb CH, Hay RJ (2008). A prospective clinical trial of a real-time polymerase chain reaction assay for the diagnosis of candidemia in nonneutropenic, critically ill adults. Clin Infect Dis.

[40] Barnes RA, White PL, Bygrave C, Evans N, Healy B, Kell J (2009). Clinical impact of enhanced diagnosis of invasive fungal disease in high-risk haematology and stem cell transplant patients. J Clin Pathol.

